# Active Methanotrophs in Suboxic Alpine Swamp Soils of the Qinghai–Tibetan Plateau

**DOI:** 10.3389/fmicb.2020.580866

**Published:** 2020-11-12

**Authors:** Yongliang Mo, Xing-e Qi, Aorui Li, Xinfang Zhang, Zhongjun Jia

**Affiliations:** ^1^College of Environmental Science and Engineering, China West Normal University, Nanchong, China; ^2^State Key Laboratory of Soil and Sustainable Agriculture, Institute of Soil Science, Chinese Academy of Sciences, Nanjing, China; ^3^School of Life Sciences, Lanzhou University, Lanzhou, China

**Keywords:** suboxic methane oxidation, nitrogen fixation, swamp soil, Qinghai–Tibetan Plateau, high-throughput sequencing, stable-isotope probing

## Abstract

Methanotrophs are the only biofilters for reducing the flux of global methane (CH_4_) emissions in water-logged wetlands. However, adaptation of aerobic methanotrophs to low concentrations of oxygen and nitrogen in typical swamps, such as that of the Qinghai–Tibetan Plateau, is poorly understood. In this study, we show that *Methylobacter-*like methanotrophs dominate methane oxidation and nitrogen fixation under suboxic conditions in alpine swamp soils. Following incubation with ^13^C-CH_4_ and ^15^N-N_2_ for 90 days under suboxic conditions with repeated flushing using an inert gas (i.e., argon), microbial carbon and nitrogen turnover was measured in swamp soils at different depths: 0–20 cm (top), 40–60 cm (intermediate), and 60–80 cm (deep). Results show detectable methane oxidation and nitrogen fixation in all three soil depths. In particular, labeled carbon was found in CO_2_ enrichment (^13^C-CO_2_), and soil organic carbon (^13^C-SOC), whereas labeled nitrogen (^15^N) was detected in soil organic nitrogen (SON). The highest values of labeled isotopes were found at intermediate soil depths. High-throughput amplicon sequencing and Sanger sequencing indicated the dominance of *Methylobacter*-like methanotrophs in swamp soils, which comprised 21.3–24.0% of the total bacterial sequences, as measured by ^13^C-DNA at day 90. These results demonstrate that aerobic methanotroph *Methylobacter* is the key player in suboxic methane oxidation and likely catalyzes nitrogen fixation in swamp wetland soils in the Qinghai–Tibetan Plateau.

## Introduction

Methane-oxidizing bacteria are the only biofilter of atmospheric methane, a potent greenhouse gas emitted from anoxic environments. It is estimated that up to 90% of anaerobically produced methane could be consumed by methanotrophs before it escapes to the atmosphere from water-logged suboxic or even anaerobic environments, such as natural wetlands, marine sediment, paddy soils, and rivers (Conrad, 2009, 2020). Although anaerobic methane oxidation plays a pivotal role in marine ecosystems (Knittel and Boetius, 2009), oxygen functions as the primary electron acceptor to facilitate methane oxidation by aerobic methanotrophs in terrestrial environments (Ho et al., 2013, 2017; Serrano et al., 2014). Aerobic methanotrophs are classified into Alphaproteobacteria (type II), Gammaproteobacteria (type I and type X), and some thermoacidophilic Verrucomicrobia guilds (Hanson and Hanson, 1996; Op den Camp et al., 2009; Knief, 2015). In the absence of oxygen, anaerobic methane-oxidizing archaea (ANME) use alternative electron acceptors to perform anaerobic oxidation of methane (AOM). These ANMEs include the euryarchaeal ANME-1, ANME-2, and ANME-3 groups. AOM processes are classified into sulfate-dependent (Boetius et al., 2000), denitrifying (Ettwig, 2010), and metal-dependent (Beal et al., 2009). AOM, according to their respective electron acceptor (i.e., sulfate, nitrate, nitrite, and metals, respectively).

The availability of electron acceptors is a key factor in shaping the diversity of aerobic methanotrophs (Fan et al., 2020). Due to the rapid depletion of oxygen in micro-aggregates with high levels of organic carbon, such as deep wetland soils, anoxic habitats could be formed in favor of ANMEs. This suggests the importance of electron acceptors other than oxygen in AOM during methane oxidation. Nevertheless, the presence of trace oxygen in shallow depths of wetland soils permits the growth of aerobic methanotrophs (e.g., *Methylobacter*). *Methylobacter* is a type I aerobic methanotroph that is pervasive in microbial soil communities where oxygen levels are between 15 and 75 μM. At oxygen levels above 150 μM, relative abundance of *Methylobacter* drops drastically (Hernandez et al., 2015). Thus, *Methylobacter* is tolerant to suboxic conditions, a key identifying feature of microaerophiles (Morris and Schmidt, 2013). Previous studies have demonstrated the role of *Methylobacter*-driven methane oxidation under anaerobic conditions using samples of Lacamas lake water (van Grinsven et al., 2020) and sub-Arctic lake sediment (Martinez-Cruz et al., 2017). However, little is known about active methane oxidizers in suboxic wetland ecosystems.

Methanotrophic nitrogen fixation by obligate types I, II, and X methanotrophs has been characterized in a number of batch cultures including *Methylomonas albus* BG8, *Methylobacter capsulatus* Y, *Methylosinus trichosporium* OB3b, and *Methylococcus capsulatus* (Bath) (Murrell and Dalton, 1983). Type II methanotrophs possess higher nitrogenase activity than type I methanotrophs (Murrell and Dalton, 1983). In complex water-flooding environments, the bioavailable nitrogen fixed by methanotrophs could partly compensate for nitrogen loss via denitrification (230 ± 60 Tg N year^–1^) (DeVries et al., 2012). Diazotrophic methanotrophs play a pivotal role in sustaining peatland productivity, as methanotrophy-induced nitrogen fixation contributes up to 40% of the nitrogen supply during peatland development (Larmola et al., 2014; Vile et al., 2014; Ho and Bodelier, 2015). It is interesting to note that ANME archaea can also fix inert gas N_2_ in marine sediments (Dekas et al., 2009, 2018). ^15^N_2_ isotope tracing is a classic method for detecting and measuring nitrogen fixation. It is a powerful method to quantify diazotrophic activity in complex environments. Although numerous studies have employed acetylene reduction assay (ARA) to measure nitrogen fixation, this method can suppress nitrogenase leading to biases in measurements (Saiz et al., 2019). Likewise, inhibition of acetylene to methane monooxygenase can underestimate the nitrogen-fixing potential by microbial methane oxidizers (Flett et al., 1975; Prior and Dalton, 1985).

The alpine wetlands in Tibet account for approximately 1% of the global CH_4_ budget from freshwater wetlands (Jin et al., 1999). The purpose of this study was to quantify the methanotrophy and diazotrophy potentials of alpine swamp soils at different soil depths. Samples were taken from the Qinghai–Tibetan Plateau. Three soil depths (i.e., 0–20, 40–60, and 60–80 cm) were studied to identify active microbes involved in methanotrophic nitrogen fixation processes in this hotspot of methane production (Hirota et al., 2004). Soil depths of 20–40 cm were not included in this incubation because soils at 40–80 cm could better represent suboxic conditions, while the surface soil of 0–20 cm was more likely subjected to high concentration of oxygen. We hypothesized the following: (1) Different soil depths possess strong methanotrophic and diazotrophic potentials under suboxic conditions. (2) Microbial-mediated processes reflect the impact of long-term environmental conditions, such as methane and oxygen concentrations. (3) Aerobic methanotrophs play a key role in oxidizing methane in swamp soils.

## Materials and Methods

### Sites and Sampling

The swamp in this study is located at Maqu, Gansu province, PR China (101°52′16″ E, 33°39′58″ N; elevation, 3450 m). This region is part of Qinghai–Tibetan Plateau with typical plateau continental climate. The annual temperature is ∼1.2°C, and precipitation is about 615 mm.

The swamp soils were flooded with water depth of 0.3–0.5 m under field conditions. The grass covers 95% the swamp, dominated by *Carex thibetica Franch*, P*otentilla anserina L.*, *Poa poophagorum Bar*, *Polygonum viviparum L.*, and *Agrostis clavata Trin*.

Swamp soils at the depth of 0–20, 40–60, and 60–80 cm were collected in June 2017 with three replications at each depth. Then soils were transported to the lab and stored at 4°C until microcosm incubation. Besides, soils for DNA extraction were stored at −80°C refrigerator.

### Microcosm Isotopic Labeling Under Suboxic Condition

For microcosm incubation of suboxic methane oxidation, fresh soils equivalent to 5.0 gram of dry weight were used. Soil samples were first placed into 120 ml glass bottles before addition of 5 ml nutrient solution contains 0.2 mM of NaCl and 30 mM of Na_2_SO_4_. The bottles were sealed by rubber stoppers and fixed with aluminum caps. Samples were pre-incubated in 4°C dark condition without shaking for 24 h so as to stabilize microbial activity and thus to reach the equilibrium of soil oxygen consumption. After pre-incubation, the headspace gas in the bottles was evacuated for 3 min by a vacuum pump which was connected to a rubber tube and sealed with three-way valve. After headspace gas evacuation, the same volume of chemically inert argon gas (Ar) was injected into the bottle to replace headspace gas and decrease oxygen concentration. After three times of argon gas replacement, the headspace gas was again evacuated, and the artificial gas consisting of ^13^C-CH_4_ (atom percent 99.9%), ^15^N-N_2_ (atom percent 99.9%) and Ar were injected into the bottles with a mixing ratio of 3:1:1 (CH_4_: N_2_: Ar), the total volume of mixture gas were approximate 360 ml to reach the over-pressed condition (3 atm.) to simulate water-logged wetland environment under field conditions. Then the stoppers were again sealed by silicone rubber. These bottles were incubated in 4°C dark condition without shaking. The temperature of 4°C is similar to field air temperature when soil samples were collected from swamps in Qinghai–Tibetan Plateau.

At day 90, gas sampling was performed in the headspace for measurement of CH_4_, CO_2_ concentrations and ^13^C-CO_2_ isotopic atom percentage. After gas sampling, the slurry headspace was flushed by Ar gas to remove residual methane which may bias the result due to aerobic methane oxidation. The isotopic atom excess of ^13^C in SOC and ^15^N in SON were then determined. Soil samples were stored at −20°C for measurement of SOC and SON. Soils for DNA extraction were stored at −80°C.

### Calculation of Methane Oxidation and Nitrogen Fixation Potentials

The headspace CO_2_ and CH_4_ concentrations were determined by gas chromatograph Agilent 7890A with flame-ionization detector (Santa Clara, United States). The ^13^C-CO_2_ isotope ratios were determined by GC-MS (MAT 253, FLASH EA Delta, Thermo Fisher Scientific Inc., Waltham, United States). The soil ^13^C and ^15^N atom isotopic percentages were measured by Isotope ratio mass spectrometer (Thermo Fisher Scientific Inc., Waltham, United States), after vacuum freeze-drying at −50°C and grinding to pass 0.15 mm sieve. The isotopic atom percentages were expressed as ^13^C/(^13^C + ^12^C) and ^15^N/(^15^N + ^14^N). Soil organic carbon and nitrogen contents were measured by C/N analyzer (Vario Max CN, Elementar, Langenselbold, Germany) after the removal of inorganic carbon by HCl acidification (0.5 mol/L). The methane oxidation potentials of ^13^C-CO_2_ and ^13^C-SOC were calculated as Eqs. 1, 2.

(1) $$Gas{\_}_{13\text{C}}=\frac{C_{gas}\times \left(A_{gas}-A_{gas0}\right)\times V_{h}\times 10^{6}}{V_{s}\times m\times t}$$

Where *Gas*____13__C_, methane oxidation potential of ^13^C-CO_2_, nmol/g dws/day; *C*_gas_, CO_2_ concentration at day 90,%; *A*_gas_, atom percent of ^13^C in CO_2_ at day 90,%; *A*_gas__0_, atom percent of ^13^C in CO_2_ at day 0,%; *V*_h_, volume of gas, mL; *V*s, molar volume of gas at standard condition, L/mol; *m*, dry weight soil, g; *t*, days of incubation.

(2) $$SOC{\_}_{13\text{C}}=\frac{C_{soc}\times \left(A_{soc}-A_{soc0}\right)\times m\times 10^{9}}{V_{s}\times m\times t}$$

Where *SOC*____13__C_, methane oxidation potential of ^13^C-SOC, nmol/g dws/day; *C*_soc_, soil organic carbon content at day 90,%; *A*soc, atom percent of ^13^C-SOC at day 90,%; *A*_SOC__0_, atom percent of ^13^C-SOC at day 0,%; *M*, molar mass of ^13^C, g/mol; *m*, dry weight soil, g; *t*, days of incubation.

Nitrogen fixation potentials were calculated as Eq. 3.

(3) $$F_{15\text{N}}=\frac{C_{SON}\times \left(A_{SON}-A_{SON0}\right)\times m\times 10^{9}}{M\times m\times t}$$

Where *F*_15__N_, nitrogen fixation potential, nmol/g dws/day; *C*_SON_, soil organic nitrogen content at day 90,%; *A*_SON_, atom percent of ^15^N-SON at day 90,%; *A*_SON__0_, atom percent of ^15^N-SON at day 0,%; *M*, molar mass of ^15^N, g/mol; *m*, dry weight soil, g; *t*, days of incubation.

### Soil Physico-Chemical Properties Analysis

Gravimetric soil water contents were determined by oven-drying method. Soil pH was measured by a pH electrode. Soil NH_4_^+^-N, NO_3_^–^-N, NO_2_^–^-N were extracted with 2 M KCl, and analyzed by AA3 continuous flow analyzer (SEAL Analytical GmbH, Norderstedt, Germany). Soil total phosphorus contents were assessed by Petra MAX element analyzer (XOS, East Greenbush, United States). A conductivity meter was used to measure soil conductivity. See soil properties in Supplementary Table S1.

### DNA Extraction, ^13^C-DNA Isolation, Quantitative PCR, and Sequencing

Soil genomic DNA was extracted using Fast DNA spin kit for soil according to the instructions by manufacturer (MP Biomedicals, Irvine, United States). The DNA quality and quantity were assessed by a spectrometer (NanoDrop Technologies, Wilmington, United States).

We isolated microbial ^13^C-DNA from the ^13^C-CH_4_ and ^15^N-N_2_ labeled total genomic DNA by ultra-centrifugation techniques as described before (Xia et al., 2011; Zheng et al., 2014). Briefly, DNA fractionations with CsCl buoyant densities ranging from 1.70 to 1.75 were obtained by centrifuging at 177 000 × *g* for 44 h in a vertical rotor (Beckman Coulter, Inc., Palo Alto, United States). Then the fractionated DNA samples were stored at −20°C for further analysis.

The population size of methanotrophs and diazotrophs were quantified by using functional gene biomarkers *pmoA* and diazotrophic *nifH* genes on Bio-Rad CFX96 Real-Time qPCR system, respectively. The primers were respectively A189f/mb661r (Bourne et al., 2001) and polF/polR (Gaby and Buckley, 2012). The qPCR reaction mixture in a total volume of 20 μL contains 0.5 μL of each forward and reverse primers, 1 μL of DNA templates, 10 μL of SYBR Premix Ex Taq, and 8 μL of sterilized water. The melting curve was established from 65 to 95°C at 0.5°C increment. The plasmids DNA containing the insert of target gene were prepared to generate standard curve by using the pEASY-T1 Cloning Kit (Beijing TransGen Biotech Co., Ltd., Beijing, China) and TaKaRa MiniBEST Plasmid Purification Kit Ver.4.0 (TaKaRa Bio Inc., Kusatsu, Japan) according to the manufacturers’ instructions. The PCR products were checked by 1% agarose gel electrophoresis for amplicon specificity.

Illumina MiSeq sequencing of bacterial 16S rRNA genes were performed by using the primer pair 515F/907R (Christner et al., 2001). The total volume of 50 μL PCR reagents contain 2 μL DNA templates, 1 μL of each primer, 25 μL of SYBR Premix Ex Taq (Tli RNaseH Plus, TaKaRa, Japan), and 21 μL of sterilized water. PCR products were checked and purified by 1.2% agarose gel electrophoresis to ensure PCR amplification specificity. Sequence libraries were constructed by using the TruSeq Nano DNA LT Sample Prep Kit Set A, sequencing was performed with MiSeq Reagent Kit v3 (600 cycles).

To increase phylogenetic resolution of active methanotrophs, the full-length of 16S rRNA genes of Archaea and Bacteria derived from labeled ^13^C-DNA (heavy fractions) were sequenced on ABI 3730 sequencing system by clone library construction (Applied Biosystems, Thermo Fisher, Carlsbad, United States). The primer pairs of 21F/1492R and 27F/1492R were employed to amplify archaeal and bacterial 16S rRNA genes, respectively (DeLong, 1992), and PCR mixture were the same as those for 515F/907R as mentioned above.

All the DNA sequences were deposited in the NCBI database under accession numbers of SRR12240017–SRR12240034 (sequence read archive for Illumina MiSeq sequences), MT798144–MT798236 (GenBank accessions for cloned archaeal sequences), and MT798237–MT798385 (GenBank accessions for cloned bacterial sequences).

### Bioinformatics Analysis

Illumina MiSeq sequencing data of 16S rRNA genes were first processed in QIIME version 1.9.1 (Quantitative Insights Into Microbial Ecology, please visit the website for detailed information)^1^, for quality control with the quality score of q25. Chimeras were identified by using usearch61 and then removed following paired ends joining, barcodes extraction and demultiplexing. Operational taxonomic units (OTUs) were classifying at the similarity of 97% (Caporaso et al., 2010). The sequences of 16S rRNA genes at the level of OTUs were directly annotated to different phyla, classes, orders, families and genera by using the open reference database (pick_open_references.py).

For 16S rRNA cloned bacterial and archaeal sequences, they were manually assembled and aligned in BioEdit 7.2^2^, yielded sequences size of 1300–1500 bp (Heijs et al., 2007). Chimeras were detected and removed in Ribosomal Database Project (RDP) classifier (see the website for more information)^3^, (Cole et al., 2009).

### Statistical Analysis

One-way ANOVA analysis in SPSS 16.0 (International Business Machines Corp., Armonk, United States) was used to assess the statistically significant difference among different treatments. Pairwise comparison was also conducted between the labeled and control treatment including ^13^C-SOC, ^13^C-CO_2_, ^15^N-SON, methane oxidation and nitrogen fixation potentials, gene copy numbers and relative abundance of taxa.

## Results

### Methane Oxidation and Nitrogen Fixation Potentials

**FIGURE 1:**
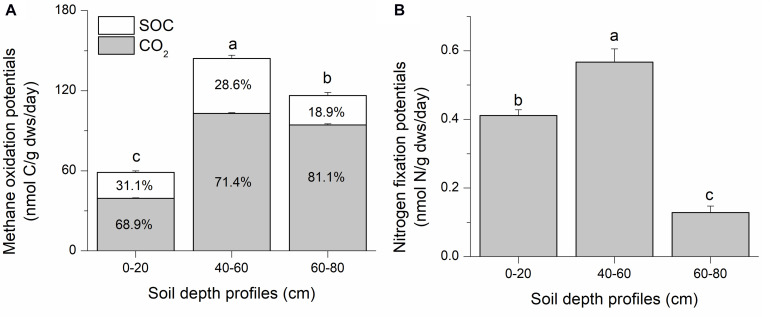
Suboxic methane oxidation **(A)** and nitrogen fixation **(B)** potentials of swamp soils collected from the Qinghai–Tibetan Plateau. Soil samples were labeled with ^13^CH_4_ and ^15^N_2_ for 90 days. Microcosm incubation was conducted with ∼6 g of fresh soil in 120-mL bottles. Suboxic conditions were generated by flushing the headspace with an inert gas (argon) three times using a vacuum pump, and a mixing ratio of 3:1:1 (CH_4_: N_2_: Ar) was achieved for incubation. In panel A, SOC and CO_2_ respectively indicate the ^13^C that were converted to soil organic carbon (^13^C-SOC) and CO_2_ (^13^C-CO_2_). They are shown as percentages within the corresponding columns. “0–20,” “40–60,” “60–80” indicate top (0–20 cm), intermediate (40–60 cm), and deep (60–80 cm) soil layers, respectively. Letters above the columns indicate significant difference (*p* < 0.05).

Methane oxidation potential was assessed as the sum of ^13^CO_2_ and ^13^C-soil organic carbon (SOC). Stable isotope tracing with ^13^C-CH_4_ and ^15^N-N_2_ indicates significant methane oxidation and nitrogen fixation potentials in all three soil depths in the Qinghai–Tibetan Plateau swamp (isotopic atom excess data are shown in Supplementary Figure S1). The intermediate soil layer (40–60 cm) had the highest methanotrophic potential (144.17 ± 3.10 nmol C/g *dws*/day), representing 2.5- and 1.2-fold increases compared to the top (0–20 cm) and deep (60–80 cm) soil layers, respectively (Figure 1A). Most of the ^13^C-derived from methane was oxidized to CO_2_ (ca. 67–81%); this value increased with soil depth. It is estimated that ∼18–31% of the ^13^CH_4_-derived carbon was incorporated into SOC via microbial biomass synthesis during methane oxidation (Figure 1A).

The highest diazotrophic potential (0.57 ± 0.04 nmol N/g *dws*/day) was observed in the intermediate soil layer, which was 4.4 and 1.4 times higher than those in deep and top soils, respectively (Figure 1B). The intermediate soils possessed the highest methane oxidation and nitrogen fixation potentials. The deep soil exhibited a higher methane oxidation potential than topsoil, but the lowest nitrogen fixation potential. The topsoil had the lowest methanotrophic potential, but with a relatively high diazotrophic potential than topsoil.

### Population Dynamics of Methanotrophic Communities

High-throughput sequencing of the total 16S rRNA genes identified five genera of methanotrophic bacteria: *Methylosinus*, *Methylocaldum*, *Methylobacter*, *Methylomicrobium*, and *Crenothrix*. In addition, one methanotrophic archaea genus, *Candidatus* Methanoperedens, was present that belonged to ANME-2d.

**FIGURE 2:**
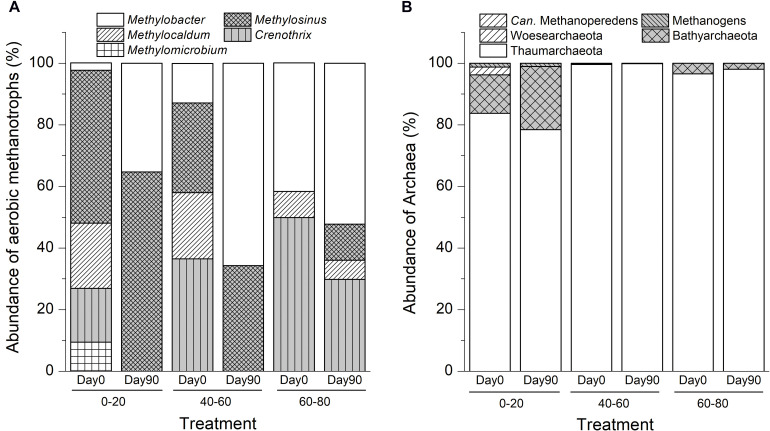
Variations in compositions of aerobic methanotrophic bacteria **(A)** and archaea **(B)** communities after suboxic methane oxidation in swamp soil profile. “0–20,” “40–60,” “60–80” indicate top (0–20 cm), intermediate (40–60 cm), and deep (60–80 cm) soil layers, respectively.

The *in situ* swamp soil methanotrophic bacteria community compositions were significantly different among different depth profiles. As shown in Figure 2A, aerobic methanotrophs comprised *Methylosinus* (50%) and *Methylocaldum* (21%) in the 0–20 cm soil layers. In the intermediate layer, methanotrophic communities were composed of *Crenothrix* (37%), *Methylosinus* (29%), and *Methylocaldum* (22%). In deep soil, the methanotrophic community was dominated by *Methylosinus* (50%) and *Methylobacter* (42%). Ammonia-oxidizing Thaumarchaeota dominated the archaeal communities in all soils, and the ANME-2d archaea *Candidatus* Methanoperedens were found in the intermediate soil with an extremely low relative abundance of 0.002% of the total microbiomes (Figure 2B).

After 90-day of incubation with methane and dinitrogen gas, the relative abundances of *Methylobacter*-like phylotypes increased by 32.9% (15-fold), 52.9% (5-fold), and 10.6% (1.3-fold) in the top, intermediate, and deep soils, respectively. The relative abundances of *Methylosinus*-like phylotypes increased by 15.1, 5.2, and 11.8% in the top, intermediate, and deep soils, respectively (Figure 2). No ANME archaea were detected in any of the soils at day 90.

### Stable Isotope Probing of Active Methanotrophs and Diazotrophs Under Suboxic Conditions

**FIGURE 3:**
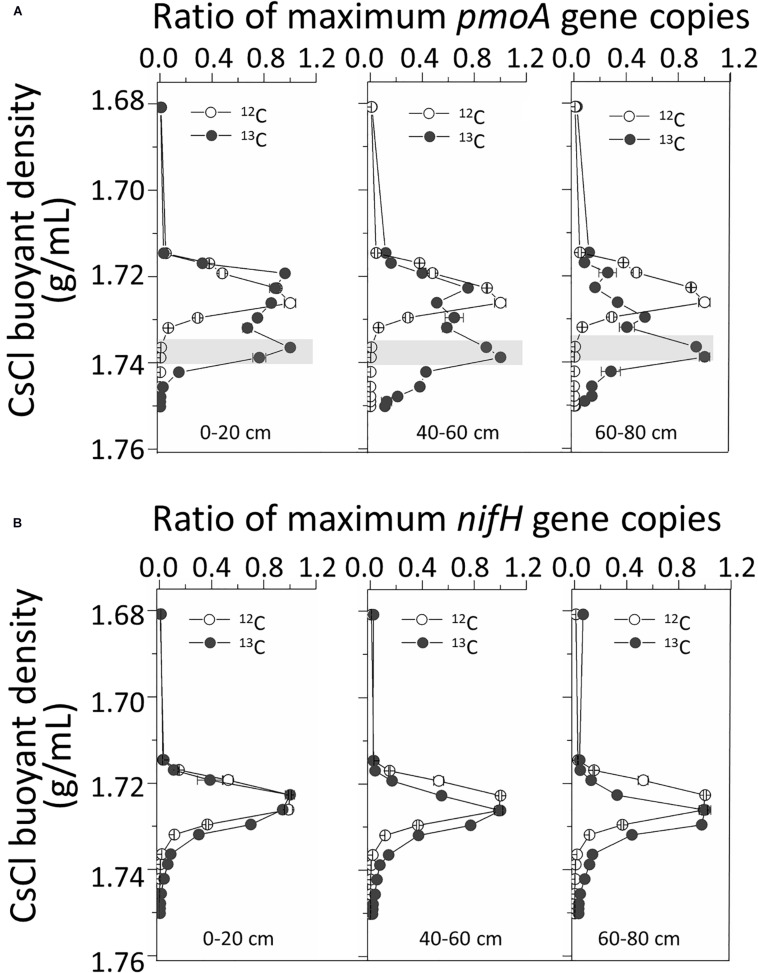
Quantitative distribution of *pmoA* **(A)** and *nifH* **(B)** gene copies across the entire CsCl buoyant density gradient of the fractionated DNA fractions from the labeled and control microcosms. “0–20,” “40–60,” “60–80” indicate top (0–20 cm), intermediate (40–60 cm), and deep (60–80 cm) soil layers, respectively.

Real-time quantitative polymerase chain reaction (qPCR) analysis of *pmoA* genes in fractionated DNA buoyant density gradients from the labeled microcosms indicated significant labeling of *pmoA* gene-carrying methanotrophs in all three ^13^C-fed soils. The *pmoA* gene copy numbers peaked at heavy ^13^C-DNA fractions with buoyant densities of ∼1.734 g/mL. For ^12^C-feed soils, the peaks were generally observed at light fractions with densities of ∼1.72 g/mL (Figure 3A). However, for *nifH* gene quantification, there were no evident peak separations between the ^13^C and ^12^C treatments (Figure 3B).

**FIGURE 4:**
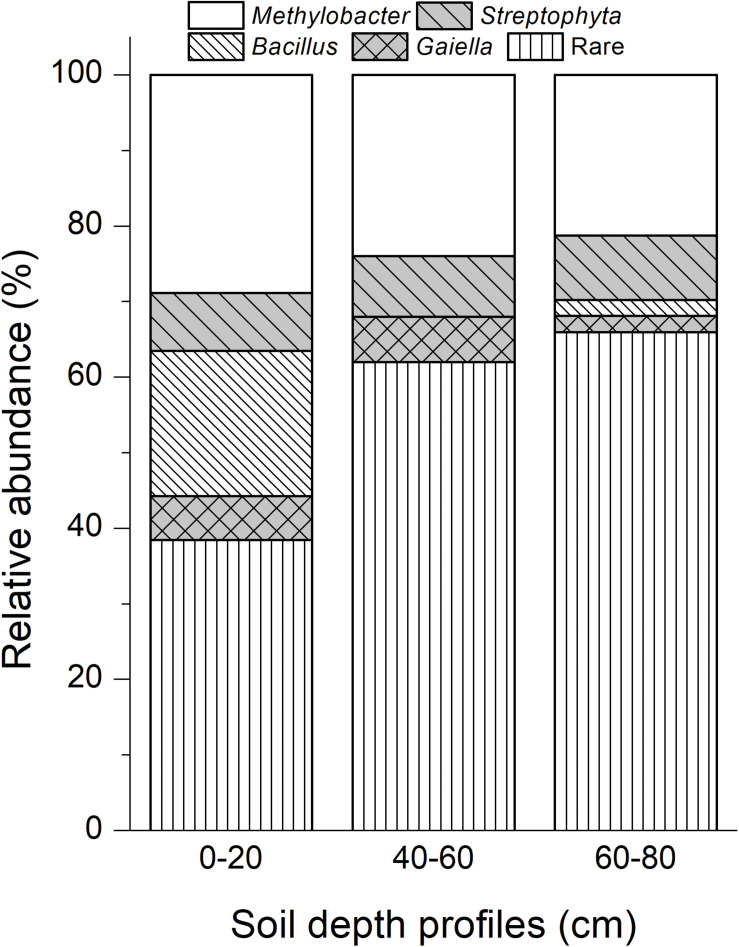
Dominant bacterial phylotypes in ^13^C-DNA based on the cloned full-length 16S rRNA genes. Rare taxa of bacteria were shown in Supplementary Table S2. “0–20,” “40–60,” “60–80” indicate top (0–20 cm), intermediate (40–60 cm), and deep (60–80 cm) soil layers, respectively.

A total of 149 cloned sequences of the full-length bacterial 16S rRNA genes in ^13^C-DNA were retrieved in addition to 94 cloned archaeal sequences from the labeled microcosms (Supplementary Table S2). Based on the analysis of the full-length 16S rRNA genes, 62 bacterial genera and 7 archaeal genera were identified (Supplementary Table S2). Among them, *Methylobacter* was the most abundant bacteria in heavy ^13^C-DNA fractions for all samples, accounting for 28.85, 24.00, and 21.28% in the top, intermediate, and deep soil bacterial clones, respectively (Figure 4).

For the archaeal community, there were no ANME archaea. However, the predominant methanogenic archaea, *Methanothrix*, accounted for 59.38, 32.26, and 38.71% of the top, intermediate, and deep soil archaea communities, respectively. *Methanosarcina* ranked second in archaea communities, with corresponding abundances of 21.88, 25.81, and 22.58% (Supplementary Table S2).

These results demonstrate that aerobic methanotrophs (i.e., *Methylobacter*) are the most active players during suboxic methane oxidation and possibly nitrogen fixation in alpine swamp soils in the Qinghai–Tibetan Plateau, whereas the role of ANMEs in this process seems to be marginal.

## Discussion

Our results show strong methane oxidation activities in swamp soils under suboxic conditions, ranging from 58 to 144 nmol C/g/day. These activities are one or two orders of magnitude lower than the methane oxidation rates in the Riganqiao peatland soils (∼3.7–4.2 μmol CH_4_/g/day) in the Qinghai–Tibetan Plateau, when an initial oxygen concentration of ∼21% was supplied (Deng et al., 2013). This suggests that oxygen limitation under suboxic conditions constrains microbial methane oxidation. Interestingly, the methane oxidation rates of subarctic *Sphagnum* mosses at low oxygen concentrations, reported by Kox et al. (2018), are 100-fold greater (i.e., 11–15 μmol CH_4/_g/day) than those in this study. This can be partly attributed to the stimulative effects of photosynthetic oxygen on methane oxidation in moss samples. Kox et al. (2018) conducted experiments under light conditions; we performed microcosm incubations under dark conditions, with lower methane oxidation potentials. Nonetheless, the suboxic methane oxidation potentials reported in this study are significantly higher than those observed under strictly anaerobic conditions, with approximately 00001–0.01 nmol/cm^3^/day in marine sediments (Sivan et al., 2007) and 0.0001–1 nmol/cm^3^/day in lake sediments (Knittel and Boetius, 2009). In addition, it is noteworthy that carbon utilization efficiency in known cultures of methanotrophs is higher than those observed in this study. The methane-derived carbon assimilation efficiencies of 19–31% were observed in soils tested, whereas higher values have been shown for aerobic *Methylomicrobium alcaliphilum* 20Z (40–50%) under 20% O_2_ (Kalyuzhnaya et al., 2013) and *Methylococcus capsulatus* (31.3–49.4%) under 16.9–19.7% O_2_ (Leak and Dalton, 1986). These aerobic efficiencies in batch cultures are comparable to those of landfill soils (29.1–39.3%) under initial O_2_ concentrations of 21 and 2.5% (He et al., 2020). However, the anaerobic efficiencies were as low as ∼1% in the sediments of the southern Hydrate Ridge in north-east Pacific (Nauhaus et al., 2007) and 1–2% in Black Sea mats (Treude et al., 2007). Future studies are required to determine the carbon utilization efficiency of methanotrophs under oxygen and nitrogen stresses in batch culture and complex environments.

The intermediate swamp soils (40–60 cm) had the highest suboxic methane oxidation potential, reflecting the impact of the long-term environmental selection of specific functional guilds by *in situ* oxygen and methane concentrations. Methane is generally considered as the sole carbon and energy source of the cell propagation of methane oxidizers (Hanson and Hanson, 1996). The deep soils are generally saturated with high concentrations of methane, which facilitates methane oxidation, resulting in higher methanotrophic potentials compared to the topsoil. Moreover, oxygen content is also a key factor influencing the methane oxidation rate by shaping different lifestyles of phylogenetically distinct methanotrophs (Ho et al., 2017). The increase in oxygen concentration under oxygen-limited conditions leads to higher methane oxidation activities (Amaral and Knowles, 1995). This may partly explain the slightly lower methane oxidation potentials of the deep soils than that of the intermediate soils.

The diazotrophic potentials of the swamp soils share similar patterns with methanotrophic potentials, i.e., with the highest potentials in the intermediate soils, implying the dependence of nitrogen fixation on methane oxidation under suboxic conditions. This is in accordance with a previous study suggesting CH_4_ oxidation-dependent N_2_ fixation in a low-nitrogen paddy soil (Shinoda et al., 2019). Methanotrophic nitrogen fixation sustains the accumulation of bioavailable carbon and nitrogen in pristine peatlands (Vile et al., 2014) and oligotrophic peatlands (Larmola et al., 2014). This dependence might be attributed to the survival strategy of methanotrophs to cope with nitrogen-deficiency during methane oxidation (Bodelier and Laanbroek, 2004). This might also be attributed to the combined effects of methane and oxygen on nitrogen fixation under long-term *in situ* conditions (Hill, 1988), although the discrepancy in the dependence between top and deep soils remains unknown.

Our results suggest that aerobic *Methylobacter*-like methanotrophs (not ANME archaea) played a pivotal role in methane oxidation in all suboxic swamp soils. The members of genus *Methylobacter* are the major driver of methane oxidation in anoxic freshwater wetland soil (Smith et al., 2018) and lake water and sediment samples (Oshkin et al., 2015; Martinez-Cruz et al., 2017; van Grinsven et al., 2020). These oxygen-limited environments might have resulted in a distinct evolutionary trajectory of methanotrophs to low oxygen stress. For instance, it has been reported that *Methylomicrobium alcaliphilum* 20Z performs fermentation-based methanotrophy with a novel pyrophosphate mediated glycolytic pathway (Kalyuzhnaya et al., 2013). Similar pathways have been found for OWC *Methylobacter* (Smith et al., 2018), *Methylomicrobium buryatense* 5GB1 (Gilman et al., 2017), *Methylomicrobium album* BG8 (Kits et al., 2015a), and *Methylomonas denitrificans* (Kits et al., 2015b) under O_2_-starvation. Functional genes responsible for dissimilatory nitrate and nitrite reduction (denitrification) were recovered from the genomes of operational *pmo* units 3 (OPU3) methanotrophic clades under oxygen-minimum zone field conditions (Padilla et al., 2017). Furthermore, the oxygenic reaction by NC10 bacteria enables the growth of aerobic methanotrophs in anaerobic environments (Ettwig, 2010), and it is unclear whether a similar metabolism exists for aerobic methanotrophs. Another adaptation strategy is the synthesis of high-affinity terminal oxidase that facilitates aerobic respiration at nanomolar oxygen concentrations (Morris and Schmidt, 2013). However, given that the initial oxygen concentration in our experiment was not zero, it is probable that ANMEs played a role in methane oxidation under strictly anaerobic field conditions.

Previous studies have identified the nitrogen-fixing potential of the *Methylobacter*, owing to the presence of *nifH* gene sequences in the strains *Methylobacter vinelandii*, *Methylobacter chroococcum*, *Methylobacter bovis*, *Methylobacter marinus*, *Methylobacter capsulatus* Y (Oakley and Murrell, 1988; Auman et al., 2001; Boulygina et al., 2002). However, the acetylene reduction method could likely lead to bias in the determination of N_2_-fixing activity of methanotrophs in batch culture and complex environments (Murrell and Dalton, 1983). This might be attributed to the inhibition of acetylene on methane monooxygenase, as this enzyme is evolutionarily related to ammonia monooxygenase, which can be effectively inhibited by acetylene (Holmes et al., 1995). Thus, the ^13^CH_4_ and ^15^N_2_ labeling methods used in this study provide strong evidences of diazotrophic activity linked to methane oxidation by *Methylobacter*-like methane oxidation bacteria, and highlight the importance of methane oxidizers on carbon and nitrogen cycling in swamp soils.

## Conclusion

Our results suggest that the aerobic methanotroph *Methylobacter* plays a key role during suboxic methane oxidation and nitrogen fixation in the Qinghai–Tibetan Plateau swamp soil. This result shows the micro-aerobic niche adaptation of obligate aerobic methanotrophs in natural suboxic environments. Further studies on the physiological metabolism and biochemical mechanisms of *Methylobacter* during the adaptation of suboxic conditions are required to better understand the global carbon and nitrogen cycles.

## Data Availability Statement

The datasets presented in this study can be found in online repositories. The names of the repository/repositories and accession number(s) can be found in the article/Supplementary Material.

## Author Contributions

ZJ conceived and designed the study, with input from XZ. YM, X-eQ, and AL conducted the experiment. YM analyzed the data and wrote the manuscript. All authors have seen and approved the final version of the manuscript.

## Conflict of Interest

The authors declare that the research was conducted in the absence of any commercial or financial relationships that could be construed as a potential conflict of interest.

## Footnotes

1 http://qiime.org

2 https://bioedit.software.informer.com/

3 http://rdp.cme.msu.edu/classifier/classifier.jsp
