# Supplementary material for: Active Methanotrophs in Suboxic Alpine Swamp Soils of the Qinghai–Tibetan Plateau
Source: Front Microbiol. 2020 Nov 12;11:580866. doi: 10.3389/fmicb.2020.580866 (PMC7689253; doi:10.3389/fmicb.2020.580866)
Supplement: Supplementary file 1 [file Data_Sheet_1.docx]

***Supplementary Materials by Mo et al., 2020***

Active Methanotrophs in Suboxic Alpine Swamp Soils of the Qinghai–Tibetan Plateau

Yongliang Mo^1, 2^, Xing-e Qi^3^, Aorui Li^3^, Xinfang Zhang^3^, Zhongjun Jia^2 *^

^1^ College of Environmental Science and Engineering, China West Normal University, Nanchong 637009, PR China

^2^ State Key Laboratory of Soil and Sustainable Agriculture, Institute of Soil Science, Chinese Academy of Sciences, Nanjing 210008, PR China

^3^ School of Life Sciences, Lanzhou University, Lanzhou 730000, PR China

*** Correspondence:**

Prof. Dr. Zhongjun Jia

jia@issas.ac.cn

Tel: +86-25-86881311; Fax: +86-25-86881000*

**The supplementary materials contain:**

1. Supplementary Table S1

2. Supplementary Table S2

3. Supplementary Figure S1

Table S1 Physico-chemical properties of swam soils collected from Qinghai–Tibetan Plateau.

| Soil depth profiles | 0–20 cm | 40–60 cm | 60–80 cm |
| --- | --- | --- | --- |
| Water content (%) | 124.00 ± 20.07 a | 81.20 ± 11.57 b | 87.00 ± 2.65 b |
| pH (H_2_O) | 5.47 ± 0.72 a | 5.33 ± 0.04 a | 5.39 ± 0.08 a |
| Conductivity (mS/cm) | 0.36 ± 0.21 a | 0.18 ± 0.02 a | 0.16 ± 0.02 a |
| SOC (g/kg) | 122.11 ± 6.41 a | 81.02 ± 22.49 b | 64.6 ± 26.37 b |
| TN (g/kg) | 6.63 ± 1.28 a | 2.60 ± 1.34 b | 2.47 ± 0.98 b |
| NH_4_-N (mg/kg) | 8.19 ± 1.23 a | 7.73 ± 0.02 a | 8.09 ± 0.69 a |
| NO_3_-N (mg/kg) | 18.54 ± 6.67 a | 10.85 ± 5.73 a | 14.34 ± 9.32 a |
| NO_2_-N (mg/kg) | 3.12 ± 0.55 a | 0.44 ± 0.13 b | 0.29 ± 0.10 b |
| TP (g/kg) | 0.86 ± 0.18 a | 0.71 ± 0.11 a | 0.61 ± 0.07 a |

**Notes**: Different letters in the same row indicate significant difference (*p*<0.05).

**Table S2** Summary for archaeal and bacterial clone library sequencing of 16S rRNA genes, their affiliations and relative abundances of these cloned sequences.

| No. | Affiliations | Clone counts | | |  | Relative abundance (%) | | |
| --- | --- | --- | --- | --- | --- | --- | --- | --- |
|  |  | 0–20 | 40–60 | 60–80 |  | 0–20 | 40–60 | 60–80 |
|  | **Archaea clones (94)** | **32** | **31** | **31** |  | **100** | **100** | **100** |
| 1 | *Methanothrix* | 19 | 10 | 12 |  | 59.38 | 32.26 | 38.71 |
| 2 | *Methanosarcina* | 7 | 8 | 7 |  | 21.88 | 25.81 | 22.58 |
| 3 | *Thermofilum* | 3 | 8 | 9 |  | 9.38 | 25.81 | 29.03 |
| 4 | *Methanolinea* | 1 | 3 | 1 |  | 3.13 | 9.68 | 3.23 |
| 5 | *Nitrososphaera* | 1 | 1 | 1 |  | 3.13 | 3.23 | 3.23 |
| 6 | *Methanocella* | 1 | 1 | 0 |  | 3.13 | 3.23 | - |
| 7 | *Caldisphaera* | 0 | 0 | 1 |  | - | - | 3.23 |
|  |  |  |  |  |  |  |  |  |
|  | **Bacteria clones (149)** | **52** | **50** | **47** |  | **100** | **100** | **100** |
| 1 | *Methylobacter* | 15 | 12 | 10 |  | 28.85 | 24.00 | 21.28 |
| 2 | *Streptophyta* | 4 | 4 | 4 |  | 7.69 | 8.00 | 8.51 |
| 3 | *Bacillus* | 10 | 0 | 1 |  | 19.23 | - | 2.13 |
| 4 | *Gaiella* | 3 | 3 | 1 |  | 5.77 | 6.00 | 2.13 |
| 5 | *Litorilinea* | 0 | 1 | 3 |  | - | 2.00 | 6.38 |
| 6 | *Haemophilus* | 0 | 3 | 0 |  | - | 6.00 | - |
| 7 | *Zhizhongheella* | 2 | 0 | 1 |  | 3.85 | - | 2.13 |
| 8 | *Clostridium* | 1 | 2 | 0 |  | 1.92 | 4.00 | - |
| 9 | *Methylosarcina* | 3 | 0 | 0 |  | 5.77 | - | - |
| 10 | *Microgenomates* | 0 | 0 | 2 |  | - | - | 4.26 |
| 11 | *Lysobacter* | 0 | 0 | 2 |  | - | - | 4.26 |
| 12 | *Methylibium* | 0 | 0 | 2 |  | - | - | 4.26 |
| 13 | *Aciditerrimonas* | 0 | 1 | 1 |  | - | 2.00 | 2.13 |
| 14 | *Defluviicoccus* | 0 | 1 | 1 |  | - | 2.00 | 2.13 |
| 15 | *Ciceribacter* | 0 | 1 | 1 |  | - | 2.00 | 2.13 |
| 16 | *Rhizobium* | 0 | 1 | 1 |  | - | 2.00 | 2.13 |
| 17 | *Brevundimonas* | 0 | 1 | 1 |  | - | 2.00 | 2.13 |
| 18 | *Ignavibacterium* | 0 | 1 | 1 |  | - | 2.00 | 2.13 |
| 19 | *Gemmatimonas* | 0 | 2 | 0 |  | - | 4.00 | - |
| 20 | *Alsobacter* | 2 | 0 | 0 |  | 3.85 | - | - |
| 21 | *Rubrivivax* | 2 | 0 | 0 |  | 3.85 | - | - |
| 22 | *Alloprevotella* | 2 | 0 | 0 |  | 3.85 | - | - |
| 23 | *Conexibacter* | 0 | 0 | 1 |  | - | - | 2.13 |
| 24 | Gp7 | 0 | 0 | 1 |  | - | - | 2.13 |
| 25 | *Solirubrobacter* | 0 | 0 | 1 |  | - | - | 2.13 |
| 26 | *Aquihabitans* | 0 | 0 | 1 |  | - | - | 2.13 |
| 27 | *Chiayiivirga* | 0 | 0 | 1 |  | - | - | 2.13 |
| 28 | *Bergeriella* | 0 | 0 | 1 |  | - | - | 2.13 |
| 29 | *Crenobacter* | 0 | 0 | 1 |  | - | - | 2.13 |
| 30 | *Geopsychrobacter* | 0 | 0 | 1 |  | - | - | 2.13 |
| 31 | Gp21 | 0 | 0 | 1 |  | - | - | 2.13 |
| 32 | *Hydrogenobaculum* | 0 | 0 | 1 |  | - | - | 2.13 |
| 33 | *Methylocapsa* | 0 | 0 | 1 |  | - | - | 2.13 |
| 34 | *Oryzihumus* | 0 | 0 | 1 |  | - | - | 2.13 |
| 35 | *Rhodoferax* | 0 | 0 | 1 |  | - | - | 2.13 |
| 36 | *Sulfuricurvum* | 0 | 0 | 1 |  | - | - | 2.13 |
|  | *Continued Table S1* | | | | | | | |
| 37 | *Verrucomicrobium* | 0 | 0 | 1 |  | - | - | 2.13 |
| 38 | *Nitrolancea* | 0 | 1 | 0 |  | - | 2.00 | - |
| 39 | Gp6 | 0 | 1 | 0 |  | - | 2.00 | - |
| 40 | *Methylomarinum* | 0 | 1 | 0 |  | - | 2.00 | - |
| 41 | *Chryseobacterium* | 0 | 1 | 0 |  | - | 2.00 | - |
| 42 | *Denitratisoma* | 0 | 1 | 0 |  | - | 2.00 | - |
| 43 | *Desulfobacca* | 0 | 1 | 0 |  | - | 2.00 | - |
| 44 | *Hydrogenispora* | 0 | 1 | 0 |  | - | 2.00 | - |
| 45 | *Propionibacterium* | 0 | 1 | 0 |  | - | 2.00 | - |
| 46 | *Caulobacter* | 0 | 1 | 0 |  | - | 2.00 | - |
| 47 | *Desulfatirhabdium* | 0 | 1 | 0 |  | - | 2.00 | - |
| 48 | *Methanomassiliicoccus* | 0 | 1 | 0 |  | - | 2.00 | - |
| 49 | *Pluralibacter* | 0 | 1 | 0 |  | - | 2.00 | - |
| 50 | *Reyranella* | 0 | 1 | 0 |  | - | 2.00 | - |
| 51 | *Saccharibacteria* | 0 | 1 | 0 |  | - | 2.00 | - |
| 52 | *Staphylococcus* | 0 | 1 | 0 |  | - | 2.00 | - |
| 53 | *Stigmatella* | 0 | 1 | 0 |  | - | 2.00 | - |
| 54 | *Sulfuricella* | 0 | 1 | 0 |  | - | 2.00 | - |
| 55 | *Massilia* | 1 | 0 | 0 |  | 1.92 | - | - |
| 56 | *Mycobacterium* | 1 | 0 | 0 |  | 1.92 | - | - |
| 57 | *Acinetobacter* | 1 | 0 | 0 |  | 1.92 | - | - |
| 58 | *Caldilinea* | 1 | 0 | 0 |  | 1.92 | - | - |
| 59 | *Pseudosporangium* | 1 | 0 | 0 |  | 1.92 | - | - |
| 60 | *Roseomonas* | 1 | 0 | 0 |  | 1.92 | - | - |
| 61 | *Tepidisphaera* | 1 | 0 | 0 |  | 1.92 | - | - |
| 62 | *Thermasporomyces* | 1 | 0 | 0 |  | 1.92 | - | - |

Notes: “-” in this table means that the relative abundance is 0%.

**Figure S1** Evidence for methane oxidation and nitrogen fixation potentials of the Qinghai–Tibetan swamp soil depth profiles under suboxic conditions. A, atom isotopic percent of ^13^C in soil organic carbon; B, atom isotopic percent of ^15^N in soil organic nitrogen; C, CO_2_ concentration in the headspace of incubation bottle; D, atom isotopic percent of ^13^C-CO_2_ in the headspace of incubation bottle. 0–20, 40–60, 60–80 indicate that soils were collected from the swamp at the depth of 0–20 cm, 40-60 cm, 60–80 cm, respectively. The asterisks above column indicate significant increase during 90 days' incubation.
